# Application of heart-rate variability in patients undergoing weaning from mechanical ventilation

**DOI:** 10.1186/cc13705

**Published:** 2014-01-23

**Authors:** Chun-Ta Huang, Yi-Ju Tsai, Jou-Wei Lin, Sheng-Yuan Ruan, Huey-Dong Wu, Chong-Jen Yu

**Affiliations:** 1 Department of Internal Medicine, National Taiwan University Hospital, Taipei, Taiwan; 2 Department of Traumatology, National Taiwan University Hospital, No. 7, Chung-Shan South Road, Taipei 100, Taiwan; 3 Graduate Institute of Clinical Medicine, National Taiwan University, Taipei, Taiwan; 4 School of Medicine, College of Medicine, Fu-Jen Catholic University, New Taipei, Taiwan; 5 Cardiovascular Center, National Taiwan University Hospital Yun-Lin Branch, Yunlin, Taiwan; 6 Graduate Institute of Epidemiology and Preventive Medicine, National Taiwan University, Taipei, Taiwan

## Abstract

**Introduction:**

The process of weaning may impose cardiopulmonary stress on ventilated patients. Heart-rate variability (HRV), a noninvasive tool to characterize autonomic function and cardiorespiratory interaction, may be a promising modality to assess patient capability during the weaning process. We aimed to evaluate the association between HRV change and weaning outcomes in critically ill patients.

**Methods:**

This study included 101 consecutive patients recovering from acute respiratory failure. Frequency-domain analysis, including very low frequency, low frequency, high frequency, and total power of HRV was assessed during a 1-hour spontaneous breathing trial (SBT) through a T-piece and after extubation after successful SBT.

**Results:**

Of 101 patients, 24 (24%) had SBT failure, and HRV analysis in these patients showed a significant decrease in total power (*P* = 0.003); 77 patients passed SBT and were extubated, but 13 (17%) of them required reintubation within 72 hours. In successfully extubated patients, very low frequency and total power from SBT to postextubation significantly increased (*P* = 0.003 and *P* = 0.004, respectively). Instead, patients with extubation failure were unable to increase HRV after extubation.

**Conclusions:**

HRV responses differ between patients with different weaning outcomes. Measuring HRV change during the weaning process may help clinicians to predict weaning results and, in the end, to improve patient care and outcome.

## Introduction

Weaning patients with respiratory failure from ventilatory support is one of the most challenging problems in intensive care. Unnecessary mechanical ventilation poses increased risk of complications to patients; however, premature liberation from mechanical ventilation may also be harmful [1]. In the past few decades, a variety of predictors have been developed to identify patients ready to breathe independently [2]. Although to date, spontaneous breathing trial (SBT) is considered the most accurate index for predicting weaning success, 15% to 20% of patients succeeding in SBT require reintubation [3,4]. The pathophysiology of weaning failure is complex and involves interaction between cardiopulmonary reserve, autonomic function, and musculoskeletal capacity [5,6]. Thus, it may be hard to assess the interplay between those factors based on a single or a few predictors.

Heart-rate variability (HRV) has been related to the balance between parasympathetic and sympathetic regulation of cardiac activity, respiration, baroreflex, and thermal regulation [7-9]. It is a noninvasive and valuable tool to characterize autonomic function and cardiorespiratory interaction [10]. The impact of mechanical ventilation on HRV has been studied in newborn babies, children with brain death, and healthy young adults placed on sedation and paralysis [11-13]. Change of HRV between different ventilator settings has also been described in a canine model [14]. Accordingly, measurement of HRV may help evaluate physiological responses to the weaning process. In a case series by Shen and associates, [15], decrease in HRV is the main finding in patients with weaning failure, and the authors suggested that change of HRV components may be a potential tool of automatically gathered parameters during ventilator weaning. However, the result has not yet been replicated in larger-scale studies. Further, change of HRV between SBT and extubation was not explored in that study.

Thus, the aim of the present study was to investigate change of HRV during the entire weaning process in patients recovering from respiratory failure. The potential predictive value of change of HRV on SBT and extubation outcomes is also evaluated.

## Materials and methods

### Study population and setting

This prospective observational study was conducted in the adult intensive care unit (ICU) of a university-affiliated hospital in Taiwan from July 2010 to November 2010. A respiratory-therapist-implemented weaning protocol was applied in the ICU. Patients who had been intubated and placed on mechanical ventilation for 24 or more hours, and were ready for their first SBT were screened for eligibility in the study. Patients were excluded if they had tracheostomies, had atrial or ventricular arrhythmia, took chronic antiarrhythmic medications, or were unable to follow verbal instructions. Patients who needed to resume ventilatory support within 30 minutes of SBT or were reintubated due to upper-airway obstruction were excluded from the data analysis.

This study was approved by the Research Ethics Committee of the National Taiwan University Hospital, and the need for written informed consent was waived.

### Weaning protocol

The weaning protocol was modified from the statement of the Sixth International Consensus Conference on Intensive Care Medicine [16]. In brief, respiratory therapists assessed the readiness for weaning and SBT on a daily basis. During the study period, SBT was conducted on a T-piece for 60 minutes, and criteria for SBT were as follows: reliable respiratory drive, stable hemodynamics, improvement of the cause of respiratory failure, positive end-expiratory pressure 8 or less cmH_2_O, fraction of inspired oxygen 40% or less, and rapid shallow breathing index <200/min/L. Patients were considered to succeed in SBT if none of the following was observed at the end of SBT: anxiety, agitation, diaphoresis, thoracoabdominal dysynchrony, respiratory rate >35 per minute, arterial oxygen saturation <90%, heart rate >140 beats per minute or sustained increase or decrease of heart rate >20%, or systolic blood pressure >180 mm Hg or <90 mm Hg [17].

If SBT was successful, the patient was immediately extubated. Extubation failure was defined as reintubation within 72 hours of extubation.

### Measurement of HRV

The HRV measurements were performed between 8 AM and 12 PM, and in this study, we used a 5-minute measurement of HRV. During the study period, drugs potentially affecting HRV were avoided if possible. Patients were maintained in a semirecumbent position and were asked to sit in a relaxed manner. After 20 minutes of familiarization with each setting, recordings were made during (a) pre-SBT period (patients were breathing on a mechanical ventilator); (b) SBT period (patients were breathing through a T-piece), and (c) postextubation period (if patients were extubated after successful SBT). In the present study, the Embletta system (Embla Systems, Broomfield, CO, USA) was used to record electrocardiography, and then the data were downloaded to a computer for analysis. The HRV analysis was performed in accordance with the Task Force recommendations [7]. Data were investigated based on frequency-domain analysis. Power-spectrum densities were calculated for very low frequency (VLF, <0.04 Hz), low frequency (LF, 0.04 to 0.15 Hz), high frequency (HF, 0.15 to 0.4 Hz), and total power (TP). Normalized units of the LF (LF%) or HF (HF%) were calculated as LF/(TP-VLF) or HF/(TP-VLF), multiplied by 100. Also, the ratios of LF to HF (LF/HF) were calculated.

### Data collection

Medical records were evaluated in detail to obtain the following information: demographic data, body mass index, acute physiology, and chronic health evaluation II score on ICU admission, comorbidities as defined in previous studies [18,19], causes of acute respiratory failure, time to first SBT, medications before the day of SBT, oxygenation status assessed by the ratio of partial pressure of oxygen in arterial blood to fraction of inspired oxygen and pre-SBT ventilator settings. Weaning parameters, including respiratory rate, minute ventilation, tidal volume, and maximal inspiratory pressure [20], were measured on the day of and before the SBT. The measured respiratory rate was then divided by the tidal volume to obtain the rapid shallow-breathing index.

### Statistical analysis

Data are presented as mean ± standard deviation or number (percentage) as appropriate. Normal distribution of VLF, TP, and LF/HF was achieved by natural logarithmic transformation and confirmed by the Kolmogorov-Smirnov test. Comparisons between groups were made by using the independent samples *t* test for continuous variables, and the χ^2^ or Fisher Exact test for categoric variables. The HRV components were analyzed by using repeated-measures analysis of variance; in case of significance, a paired *t* test was used to compare means within a group as the *post hoc* analysis. Multivariate logistic regression analysis was applied to identify independent predictors of successful SBT and extubation. Age, sex, and variables with a *P* value of <0.1 in univariate analysis were included in the multivariate model. The HRV data were dichotomized for multivariate analysis according to the best cutoffs obtained from receiver operating characteristic (ROC) curve analysis. A two-tailed *P* value of <0.05 was considered statistically significant. All statistical analysis was conducted by using statistics software (SPSS 15.0; SPSS, Chicago, IL, USA).

## Results

### Patients

**Figure 1:**
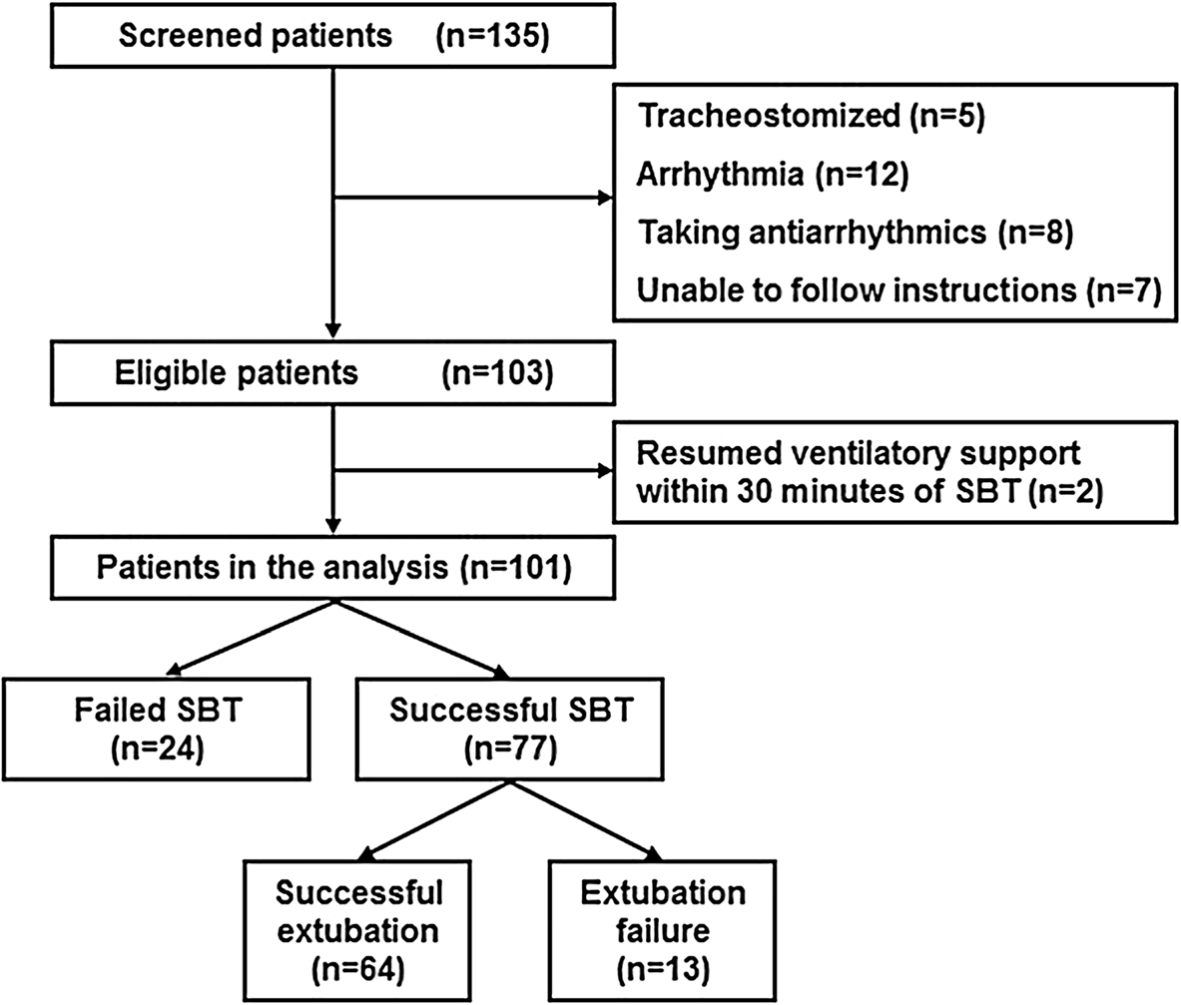
**A flow diagram of the study patients and their outcomes.** SBT, spontaneous breathing trial.

During the study period, 135 patients were screened; 32 patients were excluded for various reasons (Figure 1). Two patients who had to resume ventilatory support within 30 minutes of SBT were also excluded from the analysis because HRV measurements could not be well conducted. Of the 101 analyzed patients, 77 (76%) and 24 (24%) patients had successful and failed SBT, respectively. The demographics, baseline clinical characteristics, comorbidities, causes of acute respiratory failure, and duration of mechanical ventilation before SBT were similar between patients with successful and those with failed SBT (Table 1). Patients experiencing failure of SBT were more likely to be placed on β_2_ agonists and anticholinergics. Before SBT, no significant differences were found between the groups regarding weaning parameters, oxygenation, status and ventilator settings (Table 2).

**Table 1:** Baseline features of the study population according to the outcome of SBT

**Characteristics**	**Successful SBT**	**Failed SBT**	***P *** **value**
	**(*n*** **= 77)**	**(*n*** **= 24)**	
Age, years	65 ± 18	71 ± 16	0.186
≧65 years	45 (58)	17 (71)	0.276
Male sex	54 (70)	12 (50)	0.070
Body mass index	22 ± 4	23 ± 4	0.378
APACHE II	17 ± 6	16 ± 7	0.767
Comorbidities			
Cerebrovascular disease	16 (21)	4 (17)	0.776
COPD	19 (25)	7 (29)	0.660
Heart failure	3 (4)	1 (4)	0.999
Liver cirrhosis	4 (5)	1 (4)	0.999
End-stage renal disease	6 (8)	2 (8)	0.999
Diabetes mellitus	26 (34)	6 (25)	0.420
Hypertension	30 (39)	8 (33)	0.619
Coronary artery disease	5 (7)	3 (13)	0.391
Cause of acute respiratory failure			
Pneumonia	34 (44)	15 (63)	0.116
Sepsis	15 (20)	3 (13)	0.551
Acute exacerbation of COPD	3 (4)	1 (4)	0.999
Gastrointestinal bleeding	10 (13)	1 (4)	0.452
Lung edema	4 (5)	1 (4)	0.999
Neurologic disease	6 (8)	3 (13)	0.440
Others	5 (7)	0 (0)	0.335
Time to first SBT, hours	96 ± 103	94 ± 71	0.931
Medications			
β_2_ agonist	28 (36)	17 (71)	0.003
Anticholinergic	22 (29)	16 (67)	0.001
ACEI/ARB	8 (10)	2 (8)	0.999
CCB	27 (35)	13 (54)	0.095
β blocker	11 (14)	3 (13)	0.999

Data are presented as mean ± standard deviation or number (%).

ACEI, angiotensin-converting-enzyme inhibitor; APACHE, acute physiology and chronic health evaluation; ARB, angiotensin-receptor blocker; CCB, calcium channel blocker; COPD, chronic obstructive pulmonary disease; SBT, spontaneous breathing trial.

**Table 2:** Weaning parameters, oxygenation status, and ventilator modes before SBT based on the outcome of SBT

**Characteristics**	**Successful SBT**	**Failed SBT**	***P *** **value**
	**(*n*** **= 77)**	**(*n*** **= 24)**	
Weaning parameters			
Respiratory rate <30 per minute	70 (91)	21 (88)	0.698
Minute ventilation ≦10 L/minute	63 (82)	19 (79)	0.770
Tidal volume ≧5 ml/kg	57 (74)	15 (63)	0.276
RSBI <105/min/L	71 (92)	21 (88)	0.440
PI_max_ ≦20 cmH_2_O	73 (95)	24 (100)	0.570
PaO_2_/F_I_O_2_	318 ± 112	279 ± 101	0.128
Pre-SBT ventilator settings			
PSV	73 (95)	21 (88)	0.674
PCV	2 (3)	1 (4)	
SIMV	1 (1)	1 (4)	
VCV	1 (1)	1 (4)	

Data are presented as mean ± standard deviation or number (%).

F_I_O_2_, fraction of inspired oxygen; PaO_2_, partial pressure of oxygen in arterial blood; PCV, pressure-controlled ventilation; PI_max_, maximal inspiratory pressure; PSV, pressure-support ventilation; RSBI, rapid shallow-breathing index; SBT, spontaneous breathing trial; SIMV, synchronized intermittent mandatory ventilation; VCV, volume-controlled ventilation.

### Frequency-domain analysis of HRV

**Figure 2:**
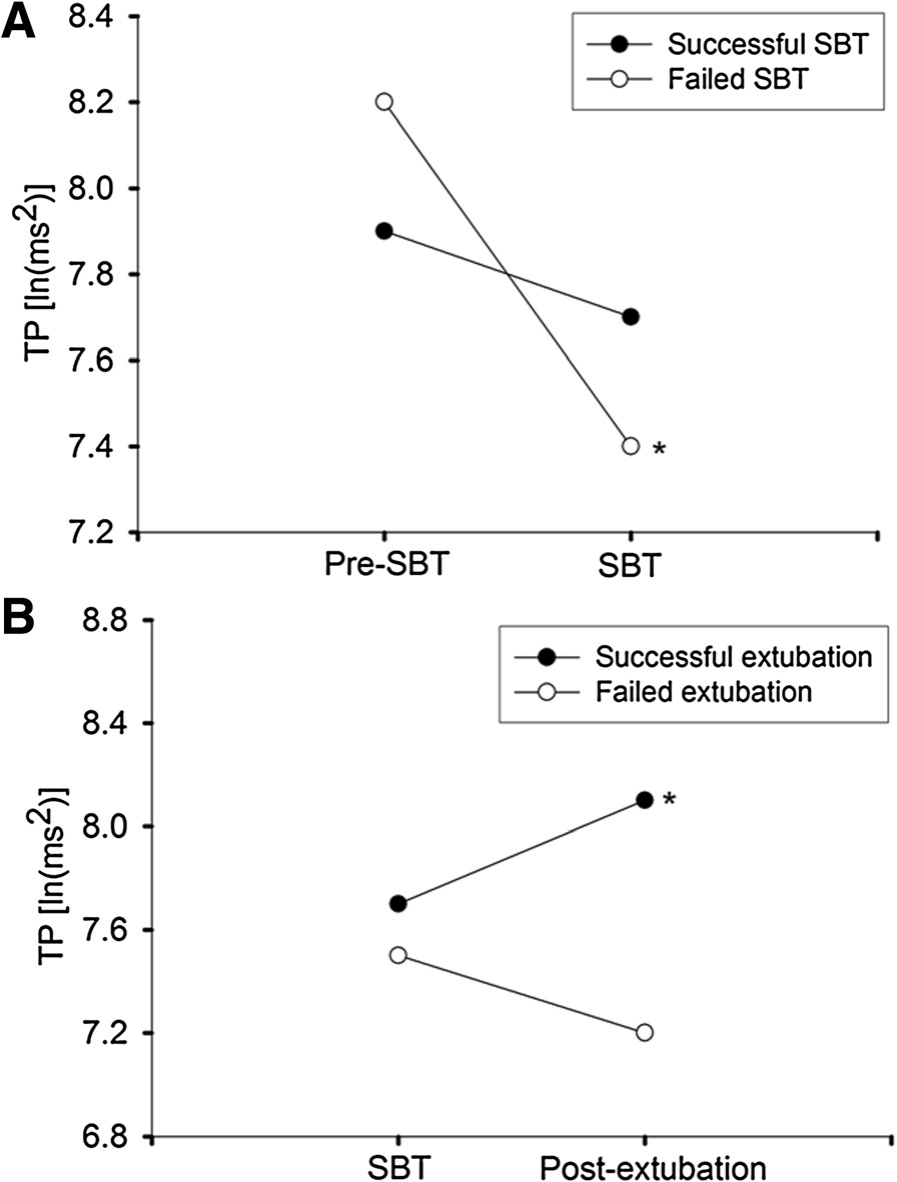
**Effect of spontaneous-breathing trial (SBT) and extubation on heart-rate variability. (A)** Patients who failed SBT had a significant decrease in total power (TP) at the change from pre-SBT to SBT periods. **(B)** Patients with successful extubation had a significant increase in TP from SBT to postextubation periods. Values are presented in means. Asterisks indicate significant changes. ln, natural logarithm.

The HRV responses to SBT in all patients are described in Table 3. Among the HRV components, significant intergroup differences were found in ln TP. Further, by comparing HRV profiles before and after SBT with the paired *t* test, significant decrease in ln TP was observed in patients with failed SBT (P =0.003) (Figure 2A). When change of ln TP (Δln TP) was dichotomized based on the cutoff established by ROC curve analysis, Δln TP ≤0.4 ln(ms^2^) was associated with an odds ratio (OR) of failed SBT of 3.1 (95% confidence interval (CI), 1.2 to 8.0). In the multivariate logistic regression model, Δln TP ≤0.4 ln(ms^2^) remained independently associated with failed SBT (Table 4).

**Table 3:** Measurement of heart-rate variability during pre-SBT and SBT periods

**Frequency-domain measures**	**Pre-SBT**	**SBT**	***P *** **value**^**a**^	***P *** **value**^**b**^
VLF [ln(ms^2^)]				
Successful SBT	6.9 (1.3)	6.3 (1.6)	0.220	
Failed SBT	6.6 (1.1)	5.5 (1.5)		
LF% (nu)				
Successful SBT	38 (20)	40 (23)	0.687	
Failed SBT	38 (20)	38 (20)		
HF% (nu)				
Successful SBT	45 (15)	44 (18)	0.775	
Failed SBT	47 (14)	45 (16)		
TP [ln(ms^2^)]				
Successful SBT	7.9 (1.0)	7.7 (1.0)	0.036	0.092
Failed SBT	8.2 (0.7)	7.4 (0.9)		0.003
LF/HF [ln(ratio)]				
Successful SBT	1.0 (0.2)	1.0 (0.3)	0.597	
Failed SBT	1.0 (0.1)	1.0 (0.2)		

^a^For comparison between successful and failed spontaneous-breathing trial groups.

^b^For comparison between pre-spontaneous-breathing trial and spontaneous-breathing trial periods within a group.

Data are presented as mean (standard deviation).

SBT, spontaneous breathing trial; VLF, very low frequency; HF, high frequency; LF, low frequency; ln, natural logarithm; nu, normalized unit; TP, total power.

**Table 4:** Multivariate logistic regression model for development of SBT failure

**Variables**	**Odds ratio (95% CI)**	***P *** **value**
Age ≧65 years	1.1 (0.3-3.6)	0.917
Female sex	3.0 (1.0-9.4)	0.054
Use of β_2_ agonist	4.0 (0.9-18.3)	0.075
Use of anticholinergic	2.9 (0.6-13.0)	0.163
Use of CCB	2.3 (0.7-6.8)	0.149
Δln TP ≤0.4 ln(ms^2^)	7.2 (2.0-25.4)	0.002

CCB, calcium channel blocker; CI, confidence interval; Δln TP, change of natural logarithmic transformation of total power from pre-SBT to SBT periods; SBT, spontaneous-breathing trial.

### Extubated patients

Among 77 patients who passed the SBT, 13 (17%) patients required reintubation within 72 hours after extubation because of hypoxemia (four patients), respiratory acidosis (four patients), respiratory distress (three patients), or hemodynamic instability (two patients). Patients with successful extubation or extubation failure had similar baseline features (Table 5). In this study, weaning parameters did not predict the outcome of extubation (Table 6). Early change of spectral components of HRV after extubation is depicted in Table 7. Significant intergroup differences in ln VLF and ln TP were observed. In successfully extubated patients, Δln VLF and Δln TP from SBT to postextubation periods showed statistically significant increase (*P* = 0.003 and *P* = 0.004, respectively); whereas, by contrast, patients for whom extubation failed were unable to increase HRV after extubation (Figure 2B). The Δln VLF and Δln TP were dichotomized based on ROC curve analysis; Δln VLF <0.1 ln(ms^2^) (OR, 3.5; 95% CI, 1.0 to 12.6) and Δln TP <0.02 ln(ms^2^) (OR, 5.6; 95% CI, 1.4 to 22.2) were significantly associated with extubation failure. After adjustment for age and sex, Δln VLF <0.1 ln(ms^2^) (OR, 3.9; 95% CI, 1.1 to 14.8) and Δln TP <0.02 ln(ms^2^) (OR, 6.6; 95% CI, 1.6 to 27.9) remained significantly related to development of extubation failure.

**Table 5:** Features of extubated patients according to the outcome of extubation

**Characteristics**	**Successful extubation**	**Extubation failure**	***P *** **value**
	**(*n*** **= 64)**	**(*n*** **= 13)**	
Age, years	64 ± 19	71 ± 15	0.247
≧65 years	36 (56)	9 (69)	0.387
Male sex	45 (70)	9 (70)	0.999
Body mass index	22 ± 5	21 ± 3	0.373
APACHE II	17 ± 6	19 ± 7	0.303
Comorbidities			
Cerebrovascular disease	14 (22)	2 (15)	0.725
COPD	16 (25)	3 (23)	0.999
Heart failure	3 (5)	0 (0)	0.999
Liver cirrhosis	3 (5)	1 (8)	0.530
End-stage renal disease	4 (6)	2 (15)	0.266
Diabetes mellitus	23 (36)	3 (23)	0.525
Hypertension	26 (41)	4 (31)	0.506
Coronary artery disease	5 (8)	0 (0)	0.582
Cause of acute respiratory failure			
Pneumonia	29 (45)	5 (39)	0.650
Sepsis	13 (20)	2 (15)	0.999
Acute exacerbation of COPD	2 (3)	1 (8)	0.430
Gastrointestinal bleeding	8 (13)	2 (15)	0.674
Lung edema	4 (6)	0 (0)	0.999
Neurologic disease	4 (6)	2 (15)	0.266
Others	4 (6)	1 (8)	0.999
Time to first SBT, hours	102 ± 109	64 ± 55	0.221
Medications			
β_2_ agonist	22 (34)	6 (46)	0.530
Anticholinergic	17 (27)	5 (39)	0.502
ACEI/ARB	7 (11)	1 (8)	0.999
CCB	24 (38)	3 (23)	0.525
β blocker	9 (14)	2 (15)	0.999

Data are presented as mean ± standard deviation or number (%).

ACEI, angiotensin-converting-enzyme inhibitor; ARB, angiotensin-receptor blocker; APACHE, Acute Physiology And Chronic Health Evaluation; CCB, calcium channel blocker; COPD, chronic obstructive pulmonary disease; SBT, spontaneous breathing trial.

**Table 6:** Weaning parameters, oxygenation status, and ventilator modes before SBT based on the outcome of extubation

**Characteristics**	**Successful extubation**	**Extubation failure**	***P *** **value**
	**(*n*** **= 64)**	**(*n*** **= 13)**	
Weaning parameters			
Respiratory rate <30/min	57 (89)	13 (100)	0.595
Minute ventilation ≦10 L/min	51 (80)	12 (92)	0.442
Tidal volume ≧5 ml/kg	47 (73)	10 (77)	0.999
RSBI <105 /min/L	59 (92)	12 (92)	0.999
PI_max_ ≦20 cmH_2_O	60 (94)	13 (100)	0.999
PaO_2_/F_I_O_2_	318 ± 115	319 ± 100	0.980
Ventilator settings before SBT			
PSV	61 (95)	12 (92)	0.592
PCV	1 (2)	1 (8)	
SIMV	1 (2)	0 (0)	
VCV	1 (2)	0 (0)	

Data are presented as mean ± standard deviation or number (%).

F_I_O_2_, fraction of inspired oxygen; PaO_2_, partial pressure of oxygen in arterial blood; PCV pressure-controlled ventilation; PI_max_, maximal inspiratory pressure; PSV, pressure-support ventilation; RSBI, rapid shallow-breathing index; SBT, spontaneous-breathing trial; SIMV, synchronized intermittent mandatory ventilation; VCV, volume-controlled ventilation.

**Table 7:** Measurement of heart-rate variability during SBT and postextubation periods

**Frequency-domain measures**	**SBT**	**Postextubation**	***P *** **value**^**a**^	***P *** **value**^**b**^
VLF (ln(ms^2^))				
Successful extubation	6.4 (1.7)	6.9 (1.2)	0.015	0.003
Extubation failure	6.1 (1.4)	5.5 (1.9)		0.187
LF% (nu)				
Successful extubation	43 (23)	43 (19)	0.783	
Extubation failure	26 (12)	27 (18)		
HF% (nu)				
Successful extubation	43 (18)	44 (15)	0.956	
Extubation failure	51 (14)	52 (15)		
TP (ln(ms^2^))				
Successful extubation	7.7 (1.0)	8.1 (0.8)	0.025	0.004
Extubation failure	7.5 (1.1)	7.2 (0.9)		0.182
LF/HF (ln(ratio))				
Successful extubation	1.0 (0.3)	1.0 (0.2)	0.954	
Extubation failure	0.9 (0.1)	0.9 (0.2)		

^a^For comparison between successful extubation and extubation-failure groups.

^b^For comparison between spontaneous-breathing trial and postextubation periods within a group.

Data are presented as mean (standard deviation).

HF, high frequency; LF, low frequency; ln, natural logarithm; nu, normalized unit; SBT, spontaneous breathing trial; TP, total power. VLF, very low frequency.

## Discussion

This is the largest study to date demonstrating the relation between change of HRV and outcomes of SBT and extubation in critically ill patients undergoing weaning from mechanical ventilation. The two major findings are that first, reduced HRV was significantly associated with SBT failure among patients with their first SBT; second, inability to increase HRV after extubation correlated with extubation failure in patients who passed SBT.

During the weaning process, the use of SBT could impose cardiopulmonary stress on certain patients. Specifically, patients for whom SBT fails are subjected to higher cardiovascular and pulmonary stress than those who succeed in the trial [21-23]. When the stress system is activated, it responds with consequent release of catecholamines due to sympathetic stimulation [24]. Decrease in HRV may reflect elevated sympathetic activity and an impairment in the physiological regulatory and adaptive mechanisms [25,26]. In line with the concepts and the findings of Shen [15], the present study showed reduced HRV in patients with failed SBT. Further, more-reduced HRV was an independent predictor of SBT failure in multivariate analysis. Continuous monitoring of electrocardiography is the routine practice in modern ICU care; thus, HRV measurements should be easily accessible and helpful in evaluating patients receiving SBT. However, real-time analysis of HRV remains challenging, because greater effective lengths of observation are required to provide better spectral resolution. Yet, in this era of rapidly advancing information technology in healthcare, we believe that this drawback will be solved in the near future, and point-of-care monitoring of HRV could be clinically feasible.

Similar to HRV, the rhythmic activity of respiration is not monotonous but is characterized by breath-to-breath variability in the tidal volume, respiratory rate, and inspiratory time of sequential breaths [27]. An inverse relation between breathing variability and respiratory loading has been observed in healthy subjects [28,29]. As expected, in patients undergoing SBT, breathing variability is greater in those successfully weaned from mechanical ventilation [30]. Breathing variability is inversely correlated with HRV in subjects free of cardiovascular and respiratory diseases [31]; however, it seems not to be the case in patients during the weaning process. Further study of simultaneously measuring breathing variability and HRV during ventilator weaning should be carried out to clarify this issue and the role it plays in predicting weaning outcomes.

One major strength in this study is that we, for the first time, assessed change of HRV after extubation. This study found significant association between increased HRV and successful extubation in patients who passed SBT. In other words, incapability to increase HRV suggested a higher probability of extubation failure. Increased HRV indicates health, fitness, and a well-functioning autonomic control mechanism [32]. Therefore, it is anticipated that patients would do better regarding the extubation outcome if they had increase in HRV after extubation. Patients requiring reintubation have a high mortality rate [1], and a direct and specific effect of extubation failure and reintubation on patient outcomes was demonstrated in a recent study [33]. Prophylactic use of noninvasive ventilation may reduce reintubation and mortality rates in patients at high risk for extubation failure [34].

However, high risk for extubation failure is difficult to define. In this study, HRV was measured shortly after extubation, and failure to increase HRV independently predicted the need for reintubation among patients being extubated after successful SBT. Early identification of at-risk patients will allow clinicians to use preventive measures such as noninvasive ventilation support, adjustment of medications, and so on, before the adverse event takes place.

In the present study, dynamic change of TP was the only recognized HRV component associated with both SBT and extubation outcomes. The TP is a net effect of all possible physiological mechanisms contributing to HRV and thus represents the overall variability of the cardiac autonomic nervous system. Of the two major components of power-spectral analysis, HF reflects modulation of parasympathetic activity; and LF indicates sympathetic modulation on the heart, although it may be also associated with parasympathetic contribution [7]. Usually, change of TP is accompanied by corresponding change in either or both of HF and LF, but we did not find such a result. This could be explained by the complex pathophysiologic process of ventilator weaning. This study enrolled a heterogeneous group of critically ill patients, and they should respond differently to the weaning process and have a variety of causes of weaning failure. Thus, the change of certain HRV components may be obscured. Further studies aiming to explore the association between HRV change and mechanisms of weaning failure will make clear the puzzle.

Other than TP, which is a reflection of total HRV, we found that change of VLF was useful in risk stratification for predicting extubation outcome. The VLF contains rhythms from several physiological variables like hormones, temperature, and vasomotion, and depends on parasympathetic outflow [10,35]. Evidence suggests that the power in this range is substantially influenced by physical activity, reflecting the activity of both parasympathetic and renin-angiotensin systems [36]. Recently, the value of VLF measurements has also been proven in risk stratification in patients with multiple organ dysfunction syndrome, because it may represent a viable interorgan communication [37]. In that study, lower VLF was significantly associated with a worse outcome. Accordingly, it is not surprising that increased VLF served as a useful index of patient well-being after extubation in the present study.

Weaning parameters have been held as a holy grail for ICU clinicians to predict weaning outcomes. However, recent studies argued against their utility in general ICU patients, neurocritical care patients, and patients with prolonged mechanical ventilation [38-40]. Similarly, weaning parameters were not associated with SBT or extubation outcomes in our work. Weaning failure is usually multifactorial; thus, weaning parameters that assess a single physiological function may have limited predictive accuracy [16,20]. Several studies have demonstrated poor specificity of these parameters for predicting weaning success [41,42]. Instead, HRV probably represents the complex interplay of multiple physiological inputs to the heart, and it would intuitively be better to predict weaning outcomes, as shown in this study.

HRV can be significantly influenced by various categories of medications; thus, the impact should be considered while interpreting HRV. The use of β_2_ agonists and anticholinergics were known to affect HRV and were more commonly seen in our patients with failed SBT [43,44]. Because all the measures in our study were completed within hours, and we compared change of HRV between study periods by using a paired *t* test, the influence of drug effects on HRV could be minimized or even eliminated. Moreover, to reduce this source of error, we tried not to administer possible confounding drugs during HRV measurements. In addition, multivariate analysis in the present study did not show an independent role of these medications on the prediction of SBT outcomes.

Certain limitations of the study must be considered. First, the results obtained must be interpreted with caution, as external factors other than SBT or extubation could have influenced the HRV values. This might hinder appropriate interpretation of the data, even though we tried hard to eliminate these intervening factors. Second, our study was conducted in a single center, and the findings may not apply similarly to other institutions, although incidence rates of SBT and extubation failure were within previously reported ranges [16]. Future multicenter studies would be helpful in this regard. Third, for the accurate measurement of HRV, we excluded patients with arrhythmia or taking antiarrhythmics, and these patients may have different HRV responses to the weaning process. More-sophisticated methods for dealing with arrhythmia during HRV analysis are needed to allow investigation of clinical implications of HRV in this specific patient population. Fourth, the HRV thresholds derived have not been prospectively validated, although the findings were pathophysiologically reasonable and predictable. Undoubtedly, validation with independent data sets will consolidate our results.

## Conclusions

Our results provide the first evidence that HRV increased after extubation in patients who succeeded in 1-hour SBT and were successfully extubated. Also, we demonstrated that patients with SBT failure had reduced HRV during the SBT period. This novel analysis of HRV data and outcomes yields a simple, noninvasive method for predicting weaning results. With prospective confirmation of these findings, this analytic method could be easily integrated into current ICU monitoring systems to help clinicians in decision making and to improve patient care. Future research could serve to explore the association of HRV change and mechanisms of weaning failure in critically ill patients.

## Key messages

• Heart-rate variability responses to the weaning process vary between patients with different weaning outcomes.

• Measurements of heart-rate variability may be of some practical value in taking care of patients weaning from mechanical ventilation.

## Abbreviations

CI: confidence interval; HF: high frequency; HRV: heart-rate variability; ICU: intensive care unit; LF: low frequency; OR: odds ratio; ROC: receiver operating characteristic; SBT: spontaneous breathing trial; TP: total power; VLF: very low frequency

## Competing interests

The authors declare that they have no competing interests.

## Authors’ contributions

CTH, YJT, and CJY conceived the study and participated in its design and coordination. CTH, JWL, SYR, and HDW participated in the data analysis and interpretation. CTH drafted the manuscript. CTH, YJT, JWL, SYR, HDW, and CJY participated in the revision of the manuscript. All authors read and approved the final manuscript.
